# Polydatin Counteracts 5-Fluorouracil Resistance by Enhancing Apoptosis via Calcium Influx in Colon Cancer

**DOI:** 10.3390/antiox10091477

**Published:** 2021-09-16

**Authors:** Hyocheol Bae, Woonghee Lee, Jisoo Song, Taeyeon Hong, Myung Hyun Kim, Jiyeon Ham, Gwonhwa Song, Whasun Lim

**Affiliations:** 1 Department of Oriental Biotechnology, College of Life Sciences, Kyung Hee University, Yongin 17104, Korea; bhc7@khu.ac.kr; 2 Institute of Animal Molecular Biotechnology, Department of Biotechnology, College of Life Sciences and Biotechnology, Korea University, Seoul 02841, Korea; cleverwhl@korea.ac.kr (W.L.); glorijy76@korea.ac.kr (J.H.); 3 Department of Food and Nutrition, College of Science and Technology, Kookmin University, Seoul 02707, Korea; js_song97@kookmin.ac.kr (J.S.); taeyeon97@kookmin.ac.kr (T.H.); 4 Department of Food and Nutrition, Sookmyung Women’s University, Seoul 04310, Korea; kimmh@sookmyung.ac.kr

**Keywords:** polydatin, colon cancer, calcium, apoptosis, 5-fluorouracil

## Abstract

Colon cancer is a disease with a high prevalence rate worldwide, and for its treatment, a 5-fluorouracil (5-FU)-based chemotherapeutic strategy is generally used. However, conventional anticancer agents have some limitations, including the development of drug resistance. Therefore, there has recently been a demand for the improvement of antitumor agents using natural products with low side effects and high efficacy. Polydatin is a natural active compound extracted from an annual plant, and widely known for its anticancer effects in diverse types of cancer. However, it is still not clearly understood how polydatin ameliorates several drawbacks of standard anticancer drugs by reinforcing the chemosensitivity against 5-FU, and neither are the intrinsic mechanisms behind this process. In this study, we examined how polydatin produces anticancer effects in two types of colon cancer, called HCT116 and HT-29 cells. Polydatin has the ability to repress the progression of colon cancer, and causes a modification of distribution in the cell cycle by a flow cytometry analysis. It also induces mitochondrial dysfunctions through oxidative stress and the loss of mitochondrial membrane potential. The present study investigated the apoptosis caused by the disturbance of calcium regulation and the expression levels of related proteins through flow cytometry and immunoblotting analysis. It was revealed that polydatin suppresses the signaling pathways of the mitogen-activated protein kinase (MAPK) and PI3K/AKT. In addition, it was shown that polydatin combined with 5-FU counteracts drug resistance in 5-FU-resistant cells. Therefore, this study suggests that polydatin has the potential to be developed as an innovative medicinal drug for the treatment of colon cancer.

## 1. Introduction

In the United States, colon cancer is one of the most rampant diseases, as the third-ranked mortality in new cancer cases. Although the mortality rate of colon cancer has gradually declined since the 1980s, it has done so at a relatively low rate, and the death rate is still high [1]. For more than 50 years, the fluoropyrimidine 5-fluorouracil (5-FU) has been commonly available as a chemotherapeutic agent to treat colon cancer [2]. However, many studies have recently reported that, in colon cancer, chemotherapy with 5-FU alone has several drawbacks, including short half-life [3]; severe systemic cytotoxic damage to the digestive organs, circulatory system, and the bioactivity of the nervous system and skin [4]; and drug resistance [5]. To overcome these limitations associated with the single chemotherapy regimen of 5-FU, combinational therapeutics that use irinotecan and leucovorin calcium have been proposed [6]. Despite the efforts to reduce the side effects of 5-FU, the addition of 5-FU to irinotecan still increased cytotoxicity; therefore, innovative adjuvant therapies need to be established to address this issue [6].

Polydatin (3,4′,5-trihydroxystibene-3-β-mono-D-glucoside) is a natural active compound obtained from the annual plant *Polygonum cuspidatum*, and used in oriental herbal medicine [7]. Pharmacological effects of polydatin have been proven in various fields of medicine. For example, polydatin has hepatoprotective effects via inhibition of inflammation [8]. In brain neuroscience, polydatin has a protective effect against neurodegenerative diseases by blocking amyloid-β peptide accumulation [9]. Moreover, for decades, it has been proven that polydatin has antitumor effects in various types of cancer. For instance, this compound suppresses the proliferation of 3D aggregates in ovarian cancer cells by inducing apoptosis [10]. In breast cancer cell lines, apoptotic cell death could be triggered by attenuating the phosphorylation of Creb induced by polydatin [11]. In addition, through the STAT3 signaling pathway, polydatin activates autophagy and leads to apoptosis in MG-63 human osteosarcoma cells [12]. Interestingly, it has been reported that polydatin reinforces the sensitivity to chemotherapy with paclitaxel in osteosarcoma cells [13]. However, whether polydatin can overcome drug resistance against the standard anticancer 5-FU drug, and the fundamental mode of action of this process, have not been elucidated yet. Considering the ability of polydatin to eradicate cancer cells and enhance chemosensitivity, it is worthwhile to investigate its primary functions and underlying mechanisms in colon cancer.

In this study, we proved that, because of its anticancer capacity, polydatin is a candidate chemotherapeutic agent against colon cancer, and can counteract the resistance to 5-FU. Using HCT116 cells that developed the resistance to 5-FU, we investigated the ability of polydatin to overcome drug resistance in combination with 5-FU. Overall, we proposed the possibility of the development promising anticancer therapy against colon cancer.

## 2. Materials and Methods

### 2.1. Chemicals and Reagents

Polydatin (Cat No: 15721), 5-FU (20 μM), and irinotecan (10 μM) were obtained from Sigma-Aldrich (St. Louis, MO, USA). The three reagents were dissolved in dimethyl sulfoxide (DMSO). The information about the antibodies used in this study is presented in a previous study [14].

### 2.2. Cell Culture

HCT116 and HT-29 cells were acquired from the Korean Cell Line Bank, and the former were cultured for at least 6 months to obtain cells resistant to 5-FU (5-FU-R), by adding progressively increasing 5-FU concentrations starting at 0.5 μM. In our prior experiment, the half-maximal inhibitory concentration (IC_50_) of cell viability to 5-FU of 5-FU-R HCT116 cells was 16.1 μM, whereas, in parental HCT116 cells, it was 1.9 μM [15]. Experiments with 5-FU-R cells were conducted in triplicate.

### 2.3. Cell Proliferation Test

Cell proliferation in response to polydatin was assessed using 5-bromo-2′-deoxyuridine (BrdU) through a Cell Proliferation ELISA kit (Cat No: 11647229001, Roche, Basel, Switzerland). The maintained cells were incubated with different doses of polydatin, or polydatin combined with chemotherapeutic agents, such as 5-FU and irinotecan, for 48 h. The treated cells were transferred into a BrdU solution for 2 h, and then in an anti-BrdU peroxidase solution for 1 h and 30 min. The relative absorbance value was rated at 370 nm and 492 nm through an ELISA reader. Experiments were conducted three times.

### 2.4. Cell Cycle Test

The allotment of cells in the cell cycle was determined using propidium iodide (PI) dye. These cells, seeded into six-well plates, were treated for 48 h. As the cells were washed, they were stained with PI and RNase A (100 µg/mL) for 30 min. Fluorescence intensity was assessed using a flow cytometer (BD Accuri C6 Plus, BD Bioscience) and BD Accuri C6 Plus software. The experiment was conducted three times.

### 2.5. Spheroid Formation

Using the hanging drop method, the spheroid for colon cancer cell lines was formed. Both cell lines were cultured for 3 days with polydatin (300 μM), or a combination of polydatin with chemotherapeutic agents, including 5-FU and irinotecan. Spheroid morphology was observed under a Leica DM3000 microscope. The relative area and density of each spheroid image were quantified with ImageJ (NIH, Bethesda, MD, USA) and ReViSP software (MathWorks, Natick, MA, USA). Briefly, the whole images acquired from the ImageJ software were modified to globular 3D images using the ReViSP software. The relative density was calculated based on the automatically set volume values. The experiment was conducted in triplicate.

### 2.6. Mitochondrial Membrane Potential (MMP) Test

The alteration of MMP was detected using a mitochondria staining kit (Cat No: CS0390, Sigma-Aldrich). The colon cancer cell lines were incubated with polydatin for 24 h, were stained using a JC-1 dye for 20 min, and finally, they were rinsed before analysis. Fluorescence levels were evaluated by a flow cytometer (BD Accuri C6 Plus, BD Bioscience). The number of cells analyzed in each dot plot was 10,000, respectively. JC-1 measurement data were compensated according to the Accuri cytometer application note. The experiment was performed in triplicate.

### 2.7. Reactive Oxygen Species (ROS) Assay

The oxidative stress was analyzed by staining the cells with 2′,7′-dichlorofluorescin diacetate (DCFH-DA, Cat No: D6883, Sigma-Aldrich) for 0.5 h at 37 °C. After staining, these cells were incubated with different doses of polydatin (0, 100, 200, and 300 μM), or polydatin (300 μM) added with a ROS inhibitor, such as N-acetyl-L-cysteine (NAC), for 1 h. Fluorescence intensity was assessed using a flow cytometer. The experiment was performed in triplicate.

### 2.8. Determination of Mitochondrial Ca^2+^ Level (Rhod-2 Assay)

The colon cancer cell lines were transferred onto six-well plates and maintained with different doses of polydatin (0, 100, 200, and 300 μM), or polydatin (300 μM) with calcium chelators, including 2-aminoethoxydiphenyl borate (2-APB; Cat No: D9754, Sigma-Aldrich), and 1,2-bis (2-aminophenoxy) ethane-*N*,*N*,*N*′,*N*′-tetra-acetic acid tetrakis (BAPTA/AM; Cat No: sc-202488, Santa Cruz Biotechnology), for 24 h. These cells were stained by Rhod-2 dye for 30 min. Fluorescence levels were estimated using a flow cytometer. The experiment was conducted in triplicate.

### 2.9. Determination of Intracellular Ca^2+^ Level (Fluo-4 Assay)

The colon cancer cells were incubated with various concentrations of polydatin (0, 100, 200, and 300 μM), or polydatin (300 μM) with calcium chelators, such as BAPTA/AM and 2-APB, for 24 h. They were then stained with Fluo-4 dye for 20 min, and the degree of fluorescence was evaluated using a flow cytometer. The experiment was conducted in triplicate.

### 2.10. Annexin V and PI Staining

To detect apoptotic cells, Annexin V and PI staining were conducted using the Annexin V apoptosis detection kit I (BD Bioscience). The cells were incubated with polydatin (0, 100, 200, and 300 μM), or co-treated with polydatin (300 μM) and NAC, or cotreated with polydatin (300 μM) and calcium chelators, including 2-APB and BAPTA/AM. These cells were incubated with Annexin V and PI for 15 min, and the degree of fluorescence was assessed using a flow cytometer. The experiment was conducted in triplicate.

### 2.11. Immunoblotting Analysis

Whole-cell proteins were extracted and normalized with Bradford reagent (Bio-Red, Hercules, CA, USA). Then, to determine the alterations of protein expression caused by polydatin or co-treatment with polydatin and 5-FU, an immunoblotting analysis was performed, as reported in a previous study [16]. Briefly, after the extracted proteins were denatured, they were isolated by SDS-PAGE, and transferred. The membrane was analyzed and quantified through chemiluminescence detection (SuperSignal West Pico, IL, USA), using the ChemiDoc Imaging system. Each protein was normalized by each total protein or α tubulin (TUBA). The colon cancer cell lines were treated with diverse concentrations of polydatin for 24 h.

### 2.12. Statistical Tests

All quantitative statistics were based on analysis of variance using the Statistical Analysis System (SAS, Cary, NC, USA) to ascertain whether the impact of polydatin on colon cancer cell lines was statistically valid. The probability value (*p*-value) below 0.05 was judged as statistically significant. All data are presented as the mean ± standard deviation.

## 3. Results

### 3.1. Polydatin Represses Colon Cancer Cells Growth and Alters Cell Distribution in the Cell Cycle

**Figure 1:**
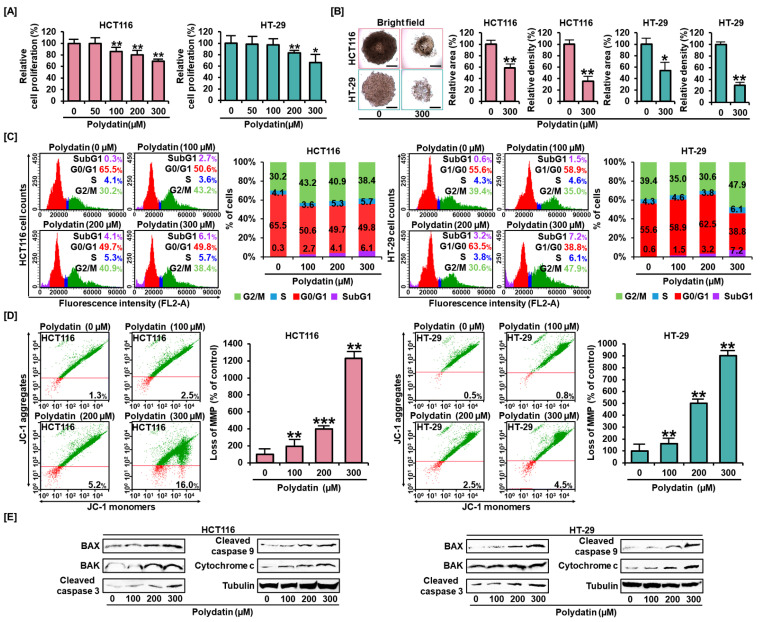
Anticancer effects of polydatin on colon cancer cells. (**A**) The proliferation analyses were conducted to determine the antiproliferative effects of polydatin (0, 50, 100, 200, and 300 µM). The relative proliferative degree of HCT116 and HT-29 cells gradually decreased with increased polydatin concentration. (**B**) The formation of spheroids was observed by Leica’s DM3000 microscope. Bright field images show the changes in spheroids morphology after treatment with 300-µM polydatin. The relative total area and density of spheroids were quantified using ImageJ. Scale bar: 100 µm. (**C**) The cell distribution in cell cycle was analyzed using PI staining. (**D**) The reduction of MMP was identified by JC-1 assay. Red represents JC-1 monomers and the green, JC-1 aggregates. The bar graphs show that the reduction of MMP was elevated with polydatin dose increasing (0, 100, 200 and 300 µM) in HCT116 and HT-29 cells. (**E**) Western blot of apoptosis-related proteins after both colon cancer cells were added with various doses of polydatin (0, 50, 100, 200, and 300 µM). The asterisks indicate significance of statistics between control and polydatin-treated groups (* *p* < 0.05, ** *p* < 0.01, and *** *p* < 0.001).

First, we estimated the antiproliferative effect of polydatin on colon cancer cell lines. The results showed a negative correlation between the decrease in HCT116 and HT-29 cell proliferation and polydatin concentration. The concentration of 300 μM of polydatin diminished cancer progression to 69% and 66% in each cell line (Figure 1A). From these results, 300 μM was set as the optimal dose and every experiment was performed using polydatin up to this concentration. Subsequently, in order to analyze the anticancer effect of polydatin in the 3D tissue environment, we conducted the spheroid formation analysis (Figure 1B). After treatment with 300 μM of polydatin, the total spheroid area formed in HCT116 and HT-29 cells was decreased to approximately 59% (*p* < 0.01) and 54% (*p* < 0.05), respectively. Moreover, this treatment lowered the relative density of construction in both cell lines to 30% and 35%, respectively (*p* < 0.01).

Based on the knowledge of polydatin’s antitumor activity, we investigated whether different concentrations of this compound could affect the cell cycle stages of colon cancer cells. The proportion of the G0/G1 phase was reduced from 65.6% to 49.9% in HCT116, and from 55.6% to 38.8% in HT-29 after treatment with polydatin. Additionally, colon cells in the S stage slightly increased in both cell lines; the percentages of G2/M cells increased moderately from 30.2% to 38.4% in HCT116, and from 39.4% to 47.9% in HT-29. Interestingly, the proportion of both cell lines in the SubG1 phase was significantly increased by polydatin (Figure 1C), suggesting the initiation of apoptosis. Overall, polydatin suppressed the proliferation of colon cancer cells and changed cell distribution in the cell cycle toward the SubG1 phase.

### 3.2. Effects of Polydatin on MMP and Apoptosis in Colon Cancer Cells

Based on the previous results demonstrating that polydatin treatment can shift the cell cycle toward the SubG1 phase, we further and comprehensively examined the cause of this alteration. First, we investigated mitochondrial dysfunction by measuring MMP. In colon cancer cell lines, the depolarization of the mitochondrial membrane was reinforced by polydatin, depending on its concentration. The ratio of JC- monomers to JC-1 aggregates soared sharply from 1.3% to 16% in HCT116, and from 0.5% to 4.5% in HT-29 cells in response to polydatin (Figure 1D). Then, we analyzed the levels of apoptosis-related proteins to confirm the impact of polydatin on apoptosis in colon cancer. Polydatin generally increased the levels of apoptosis-related proteins in both cell lines; therefore, we inferred that this compound caused apoptosis in colon cancer cells (Figure 1E).

### 3.3. Polydatin Mediates Apoptotic Cell Death through ROS Production

**Figure 2:**
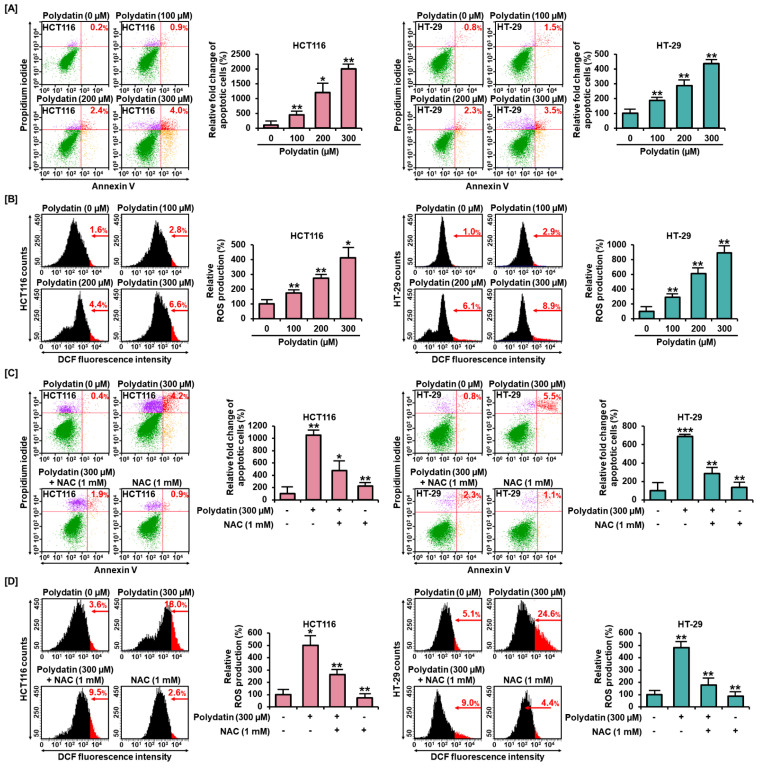
ROS production and apoptosis in response to polydatin in HCT116 and HT-29. (**A**) Apoptotic HCT116 and HT-29 treated with polydatin were evaluated using Annexin V and PI dyes. The red in the dot plot indicates late apoptotic cells. (**B**) ROS generation was determined using dichlorofluorescin (DCF) fluorescent dye by flow cytometry. (**C**) With or without ROS scavenger, the apoptotic cells induced by polydatin were stained using Annexin V and PI dyes. (**D**) The effects of polydatin ROS generation with or without NAC in HCT116 and HT-29 cells were analyzed by flow cytometry. The asterisks indicate statistical significance of the effect of treatment (* *p* < 0.05, ** *p* < 0.01, and *** *p* < 0.001).

Based on the assumption that polydatin could induce apoptosis in colon cancer cells, we further investigated the impact of this process by performing an Annexin V and PI staining assay. In response to 300 μM of polydatin, the relative cell death significantly increased by about 2000% in HCT116 (*p* < 0.01) and 437% in HT-29 (*p* < 0.01) compared to the control (0 μM) (Figure 2A). Then, we ascertained the cellular mechanism of apoptosis triggered by polydatin. Excessive ROS generation could induce apoptosis in various cancer cells [17]; therefore, we investigated the generation of ROS products in response to polydatin. In both cell lines, after treatment with polydatin, it was shown that the relative ROS production increased gradually with increasing concentrations of the compound (Figure 2B). Then, to reveal the relationship between ROS production and the apoptosis resulted from polydatin treatment, we conducted the Annexin V and PI and ROS assays with or without NAC, which is a ROS scavenger. NAC significantly restored the ROS production induced by polydatin from 500% to 264% in HCT116 cells, and from 482% to 176% in HT-29 cells (Figure 2D). In addition, the combination of NAC with polydatin effectively reduced the apoptotic cells from 1050% to 475% in HCT116, and from 688% to 288% in HT-29, in comparison with the treatment using polydatin alone (Figure 2C). Overall, it was shown that polydatin could induce apoptosis through oxidative stress in colon cancer cells.

### 3.4. Dysregulation of Ca^2+^ Leads to Cell Death in Colon Cancer Cells

**Figure 3:**
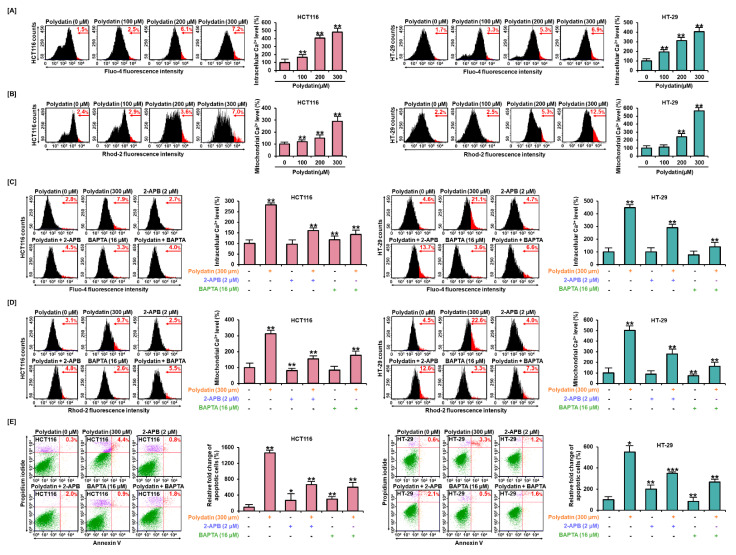
Polydatin caused the disturbance of calcium regulation and apoptosis in HCT116 and HT-29 cells. (**A**) In response to polydatin, the cytosol calcium concentrations in colon cancer cells were evaluated by flow cytometry analysis with Fluo-4 dye. (**B**) The colon cancer cells treated with polydatin were stained with Rhod-2 dye to analyze mitochondrial calcium concentrations. (**C**,**D**) Effects of polydatin with specific calcium-channel inhibitors, 2-APB (IP3R), BAPTA/AM (intracellular calcium), (**C**) cytosol, and (**D**) mitochondrial calcium levels in HCT116 and HT-29 cells. (**E**) Effects of polydatin with each calcium-channel inhibitor on apoptosis in HCT116 and HT-29 cells by Annexin V and PI staining. The asterisks indicate statistical significance of the effect of treatment (* *p* < 0.05, ** *p* < 0.01, and *** *p* < 0.001).

To confirm the impact of polydatin on Ca^2+^ regulation in colon cancer, we investigated intracellular and mitochondrial Ca^2+^ levels by performing a Fluo-4 assay and a Rhod-2 assay, respectively. Results showed that polydatin increased both intracellular and mitochondrial Ca^2+^ levels in both cell lines in a dose-dependent manner. The treatment with 300 μM of polydatin could markedly elevate the cytosolic Ca^2+^ levels by 480% and 405% (*p* < 0.01) in HCT116 and HT-29 cells, respectively (Figure 3A). In addition, mitochondrial Ca^2+^ concentrations sharply increased by 292% and 586% (*p* < 0.01), respectively (Figure 3B). Next, to demonstrate the response of calcium in polydatin-induced apoptosis, BAPTA/AM (the calcium chelating agent) and 2-APB (the inhibitor of inositol 1,4,5-trisphosphate (IP3) receptors) were used to modify the free Ca^2+^ levels. In both cell lines, the free Ca^2+^ levels were increased by polydatin in cytosol up to 282% and 449%, and were effectively restored when 300 μM of polydatin was combined with 2-APB or BAPTA/AM (Figure 3C). Similarly, the increased mitochondrial Ca^2+^ levels were attenuated up to 313% and 502% in each cell line by 2-APB and BAPTA/AM (Figure 3D). Moreover, based on these results, we detected colon cancer cells undergoing apoptosis induced by polydatin, with or without 2-APB or BAPTA/AM. In line with previous data, it was observed that this apoptosis induced by polydatin was drastically diminished by the combination with 2-APB or BAPTA/AM (Figure 3E). Overall, polydatin altered calcium regulation processes, consequently resulting in the apoptosis of colon cancer cells.

### 3.5. Signaling Pathways Associated with the Anticancer Effects of Polydatin in Colon Cancer Cells

**Figure 4:**
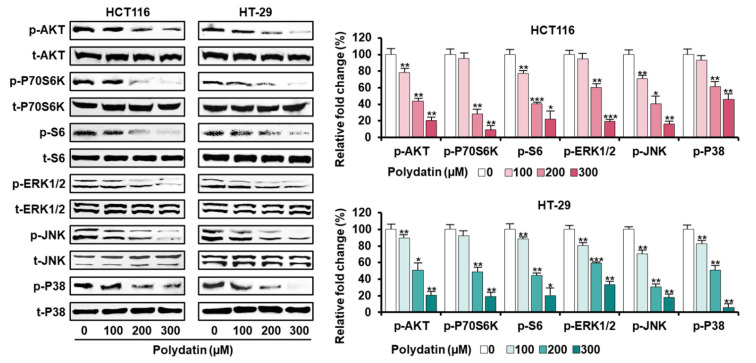
Dose-dependent inhibitory effects of polydatin on MAPK and PI3K/AKT signaling pathway in HCT116 and HT-29 cells. Each protein’s phosphorylation level was quantified with each total protein. The asterisks signify statistical variation among polydatin-treated and vehicle-treated groups (* *p* < 0.05, ** *p* < 0.01, and *** *p* < 0.001).

**Figure 5:**
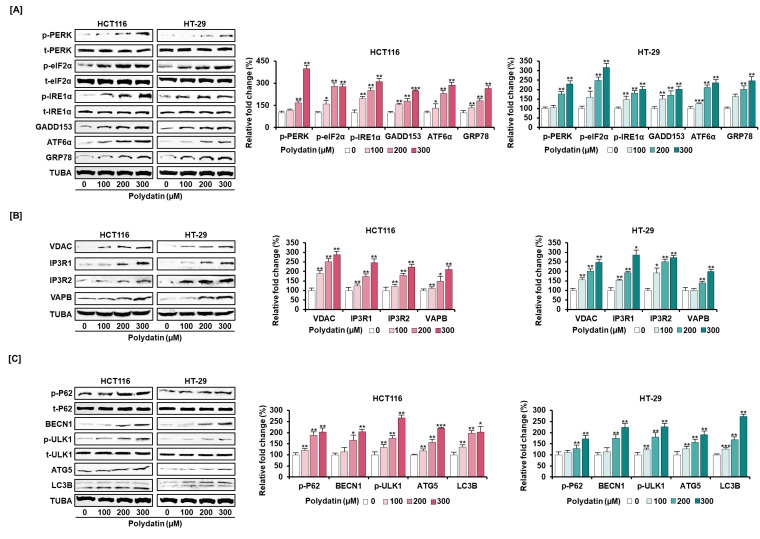
Dose-dependent stimulatory effects of polydatin on protein levels related to anticancer ability. (**A**) The protein expression of ER stress inducers—PKR-like ER-resident kinase (p-PERK), inositol-requiring enzyme 1α (IRE1α), and activating transcription factor 6α (ATF6α), ER stress sensor—eukaryotic translation-initiation factor 2α (p-eIF2α), and growth arrest and DNA damage-inducible gene 153 (GADD153), and the upregulation protein of ER stress sensor—glucose-regulated protein 78 (GRP78), when HCT116 and HT-29 cells were treated with diverse concentrations of polydatin. (**B**) Effects of polydatin on ER-mitochondria-tethering proteins (VDAC, IP3R1, IP3R2, and VAPB) in HCT116 and HT-29 cells by Western blotting. (**C**) The protein levels of phosphorylated P62 (p-P62), beclin-1 (BECN1), phosphorylated UNC-51-like kinase 1 (p-ULK1), autophagy-related 5 (ATG5), and microtubule-associated proteins 1A/1B light chain 3B (LC3B) implicated in autophagy. The expression of each protein was normalized compared with TUBA or each total protein. The asterisks mean statistical variation between polydatin-treated and DMSO-treated groups (* *p* < 0.05, ** *p* < 0.01, and *** *p* < 0.001).

In view of the anticancer effects, we confirmed the signaling transduction induced by polydatin. First, the activities of PI3K/AKT and MAPK signaling proteins (involved in cell survival) in response to polydatin were evaluated. As illustrated in Figure 4, polydatin suppressed the protein phosphorylation associated with PI3K/AKT and MAPK in colon cancer. Then, we investigated the levels of endoplasmic reticulum (ER) regulatory proteins to determine the effects of polydatin on ER stress. Overall, the expression degrees of ER stress-related factors were intensified by polydatin in both cell lines (Figure 5A). As we had previously shown that polydatin engaged in the disturbance of calcium homeostasis in colon cancer, the communication between ER and mitochondria was investigated by measuring the activity of ER-mitochondria tethering proteins. In both cell lines, the inositol 1,4,5-triphosphate receptor (IP3R)-voltage-dependent anion channel (VDAC) calcium regulation, and the expression of the vesicle-associated membrane and protein-associated protein B/C (VAPB) were upregulated by polydatin in ER-mitochondria contact sites (Figure 5B). Based on this result, we next investigated the protein levels related to autophagy regulation, because the intracellular reactions in ER-mitochondria contact sites are closely associated with the regulation of autophagy [18]. The Western blot analyses illustrated that polydatin enhanced autophagosome formation in colon cancer cell lines (Figure 5C). Altogether, our data indicate that polydatin-regulated signaling cascades could play a crucial role in cell survival, ER stress, and autophagosome formation, eventually resulting in anticancer effects in colon cancer.

### 3.6. Synergetic Effects of Polydatin and Standard Anti-Tumor Drugs on Colon Cancer

**Figure 6:**
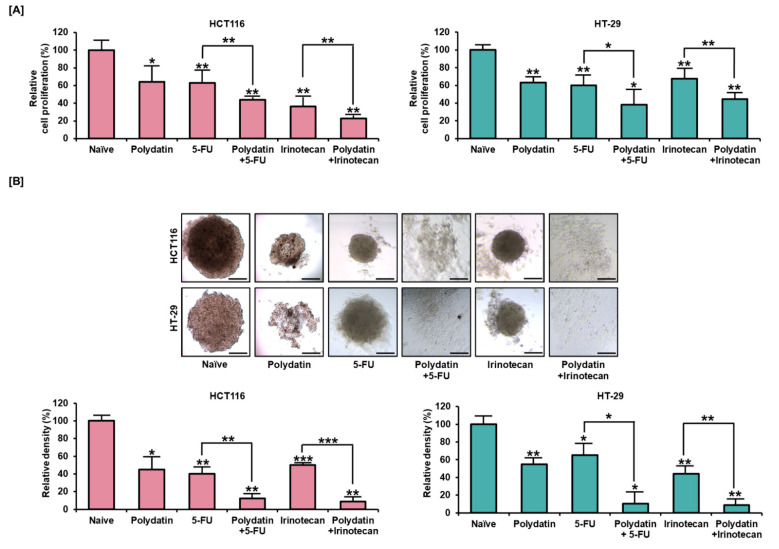
The synergetic effects of polydatin when added with accepted anticancer drugs, 5-FU, and irinotecan. (**A**) The suppressive effects of polydatin on the proliferation of colon cancer cells when combined with 5-FU and irinotecan. (**B**) The spheroids of HCT116 and HT-29 were observed by cotreating polydatin with anticancer drugs. The relative density of spheroid formed in colon cancer cells was calculated using ImageJ. Scale bar: 100 µm. The asterisks symbolize statistical differences among diverse groups (* *p* < 0.05, ** *p* < 0.01, and *** *p* < 0.001).

The antiproliferative effect of polydatin was evaluated in combination with the widely used anticancer drugs known as irinotecan or 5-FU. As shown in Figure 6A, polydatin, used in combination them, significantly attenuated the proliferation of colon cancer cells. Moreover, we confirmed that the polydatin treatment added with 5-FU or irinotecan could further hinder spheroid formation in 3D culture. In HCT116 cells, polydatin reduced the total spheroid density from 40% to 12% (*p* < 0.01) when combined with 5-FU, and from 50% to 9% (*p* < 0.001) when combined with irinotecan. In HT-29 cells, polydatin also decreased the relative spheroid density when combined with 5-FU or irinotecan from 65% to 10% (*p* < 0.05) and from 44% to 9% (*p* < 0.01), respectively, as indicated in Figure 6B. Our results suggest that a synergetic effect is produced by the combination of polydatin with standard anticancer drugs in colon cancer.

### 3.7. Polydatin Mitigates the Development of Resistance to 5-FU in Colon Cancer

**Figure 7:**
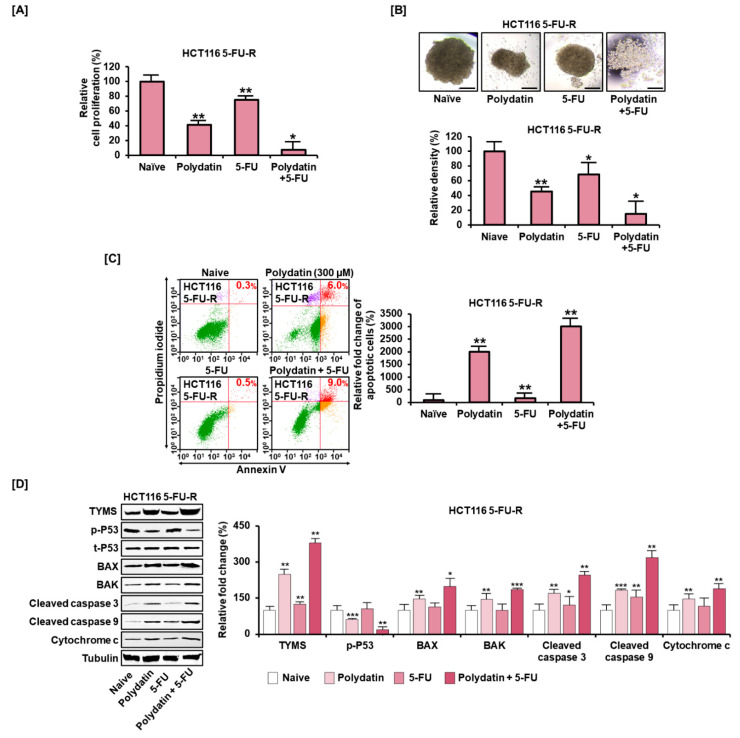
The potency of polydatin to assuage resistance of 5-FU in colon cancer cells. (**A**) Antiproliferative effect of polydatin in 5-FU-R cells with or without 5-FU. (**B**) Effect of 72-h treatment with a combination of polydatin and 5-FU on spheroids formation in 5-FU-R cells. Scale bar: 100 µm. (**C**) Apoptotic effect of polydatin in 5-FU-R cells with or without 5-FU, as elucidated by Annexin V and PI staining. (**D**) In 5-FU-R cells, effects of polydatin on the protein expression of TYMS and apoptosis-related factors with or without 5-FU, as determined by Western blotting. The asterisks symbolize significant differences among various groups (* *p* < 0.05, ** *p* < 0.01, and *** *p* < 0.001).

To determine the impact of polydatin in overcoming 5-FU resistance in colon cancer cells, 5-FU resistant cells were established by adding 5-FU to HCT116 and by increasing its concentration gradually for a long period; then, these cells were named 5-FU-resistant (5-FU-R). They exhibited elevated resistance to the antiproliferative effect of 5-FU. However, polydatin reduced the proliferation of 5-FU-R cells more effectively than the addition of 5-FU alone did, and the effectiveness was reinforced from 75% to 7% when polydatin was combined with 5-FU (Figure 7A). Similarly, the relative spheroid density in 5-FU-R cells was significantly reduced from 69% to 15% when polydatin was combined with 5-FU, compared to the treatment using 5-FU alone (Figure 7B). Moreover, polydatin markedly increased apoptosis in 5-FU-R cells by 2000% (Figure 7C). Subsequently, we assessed whether these effects on 5-FU-R cells were correlated with the activity of thymidylate synthase (TYMS), a target 5-FU protein, and apoptosis-related factors (BAX, P53, BAK, cleaved caspase 3, cleaved caspase 9, and cytochrome *c*) by Western blot analysis. Overall, 5-FU alone did not significantly alter the expression of these proteins in 5-FU-R cells. However, when polydatin was combined with 5-FU, these protein levels were upregulated, except for the phosphorylation of P53 (Figure 7D). These results imply that polydatin may have a potential to alleviate induced 5-FU resistance in colon cancer.

## 4. Discussion

The intracellular mechanisms associated with the antitumor effect and chemosensitivity of polydatin in colon cancer cells have not been clearly established yet. In our research, we confirmed that polydatin has suppressive effects on cell growth and alters cell distribution in the cycle of colon cancer cells. In addition, polydatin induces mitochondrial dysfunction through the loss of MMP and generation of ROS products. Moreover, it disturbs calcium homeostasis in cytosol and mitochondria and leads to apoptosis in colon cancer; also, it attenuates protein phosphorylation associated with the PI3K/AKT and MAPK signaling pathways. Conversely, polydatin activates the pathway of caspase cascades and the proteins involved in ER stress, ER-mitochondria tethering, and autophagosome formation in colon cancer cells. Overall, we demonstrated the antitumor effectiveness of polydatin in boosting chemosensitivity to the conventional 5-FU anticancer drug. Therefore, these results indicate that polydatin could be used as an alternative chemotherapeutic drug against colon cancer.

Many recent studies reported that, as therapeutic agent, polydatin has an apoptotic effect in various types of cancer. For example, it induces apoptotic cell death in human nasopharynx cancer cells through ROS-mediated ER stress and mitochondria defectiveness [19]. Additionally, in hepatocellular carcinoma cells, polydatin represses proliferation and induces apoptosis by blocking AKT phosphorylation [20]. It also causes cell cycle arrest and regulates the activity of *BCL-2* and *BAX* in lung cancer cell lines [21]. Similarly, we also present evidence of mitochondrial dysfunction, blocking of PI3K/AKT and MAPK signaling transduction, and stimulation of apoptosis-related proteins. These effects clearly indicate that polydatin could trigger apoptosis in colon cancer in various ways.

The regulation of intracellular calcium level is considered an essential cellular activity associated with the cell cycle, proliferation, and apoptosis in diverse types of cancer [22]. For decades, many studies have suggested that an elevation of the cytosolic or mitochondrial calcium level could induce apoptosis. For instance, apoptosis was observed in human hepatoma cells after the continuous increase in intracellular calcium level [23]. In breast cancer cells lines, apoptosis was induced through cytosolic calcium overload, and was verified by the overexpression of Bax [24]. Similarly, in thyroid carcinoma cells, interference in mitochondrial calcium regulation triggered caspase stimulation and resulted in apoptosis [25]. Additionally, calcium signaling can influence ROS generation or MMP, which are other causes of apoptosis. For example, it was shown that, in prostate cancer cells, the surge in calcium concentration generates ROS and activates caspase 3, which leads to apoptosis [26]. Followed by calcium overload, the increase of ER stress—proven by the raised levels of ER stress markers, GADD153 and GRP78—promotes the depolarization of the mitochondrial membrane in human cervical cancer cells [27]. The main targets of calcium signals, which derive from ER, are mitochondria. These influxes of calcium are attributed to the presence of mitochondria-associated ER membranes (MAMs). Therefore, when it comes to apoptosis resulting from the disruption of calcium homeostasis between mitochondria and cytosol, MAMs are regarded as key structures [28]. In this experiment, we used two calcium modulators, BAPTA/AM and 2-APB, to elucidate the mechanisms linking polydatin to calcium regulation. BAPTA/AM is a specific calcium chelator that can bind up to two calcium ions using four functional carboxylic acid groups [29,30]. The main targets of 2-APB are IP3Rs, which block the calcium release derived from IP3 [31]. In the present study, the abundance of cytosolic and mitochondrial calcium levels was detected after polydatin treatment in HCT116 and HT-29 cells. Additionally, we confirmed increases of protein expression located in MAMs and in the mitochondrial membrane, including VDAC, IP3Rs, and VAPB. Moreover, the increasing mitochondrial and cytosolic calcium levels induced by polydatin were attenuated by the addition of BAPTA/AM or 2-APB. We clarified the apoptotic effects of polydatin showing that apoptosis was enhanced despite the presence of these calcium regulators. Therefore, our hypothesis that polydatin could induce apoptosis through calcium regulation in colon cancer cells is valid.

Another intracellular event causing the disruption of calcium homeostasis is ER stress, which also leads to cellular dysfunction and apoptosis once it enters irreversible stages [32,33]. Various intracellular physiological alterations occur in ER stress conditions. Firstly, under prolonged conditions of perturbation of ER functions caused by the accumulation of unfolded proteins, various processes can be induced, such as the unfolded protein response (UPR) and well-organized intracellular reactions, to restore protein folding homeostasis. The UPR is mainly associated with three ER-localized proteins—PERK, IRE1α, and ATF6—which are retained in their inactive states by interacting with GRP78 [34]. mRNA translation is inhibited to protect ER from an overflow of newly synthesized proteins. PERK and its downstream proteins, including eIF2α and GADD153—known as C/EBP homologous protein (CHOP)—are responsible for the overall process of this translational inhibition [33,35]. A previous study has already proven that polydatin enhances apoptosis of human cancer cells through the increase of ER stress, which is identified by the increase in CHOP [19]. Secondly, the increasing expression level of the BAX and BAK proteins could also be associated with the process of ER calcium-mediated apoptosis. The temporary overexpression of these proteins stimulates the release of ER calcium, resulting in an increase in mitochondrial calcium, and eventually, these signals induce the release of cytochrome *c* [36]. For example, increased ER stress, confirmed by the higher expression levels of GRP78 and phosphorylated eIF2α (p-eIF2α), leads to apoptosis in human adenoid cystic carcinoma cell lines [37]. Herein, we observed the escalation of phosphorylated PERK (p-PERK) and eIF2α, as well as the activation of IRE1α, ATF6, GRP78, and GADD153 after polydatin treatment. Additionally, the increases in BAX, BAK, and cytochrome *c* were detected by Western blotting analysis. Therefore, ER stress induced by polydatin leads to cell death through the release of pro-apoptotic factors. Third, during ER stress response, the activation of autophagy is triggered by several molecules associated with ER stress signals [38]. Autophagy is comparable to ER stress in that it works as a double-edged sword and produces either a protective or apoptosis-promoting reaction [39]. Recently, the strategies targeting autophagy have started to be regarded as prospective methods for cancer therapy, as they suppress or promote autophagy-associated proteins. In head and neck carcinoma, the activation of autophagy through upregulation of LC3B and BECN1 produces a tumor suppressive effect [40,41]. Many experts in diverse fields of medicine have reported that polydatin has protective effects against injury of the heart, liver, and other organs, through the regulation of autophagosome formation. For example, polydatin has a powerful effect on post-treatment in C57BL/6 mice which have undergone heart injury through stimulation of autophagy. In the cell line model of non-alcoholic steatohepatitis (NASH), polydatin mitigates lipid accumulation and restores lysosomal activity via autophagic flux. Moreover, polydatin ameliorates blood vessel lesions in atherosclerotic mice by rehabilitating autophagy [42,43,44]. Our immunoblotting results suggest that polydatin stimulates autophagy-related proteins, including phosphorylated ULK1, BECN1, ATG5, and LC3B, and has a protective effect against colon cancer. Although the explicit molecular mechanisms of how polydatin affects the formation of autophagosomes are not entirely understood, our results reveal that polydatin regulates autophagy induced by ER stress in colon cancer cells. Overall, polydatin promotes the perturbation of calcium homeostasis induced by ER stress response.

In the present study, we have evidence to substantiate the hypothesis that polydatin has synergetic effects in combination with 5-FU, and the potential to counteract the resistance to conventional anticancer drugs, such as irinotecan and 5-FU, in colon cancer. The expression levels of TYMS, a main target protein for 5-FU, and phosphorylated P53 (p-P53) closely correlate with the resistance to 5-FU. The attenuation of TYMS and reinforcement of p-P53 are utilized as a strategy to enhance the chemosensitivity of colon cancer [45,46]. However, our results revealed that TYMS and p-P53 were increased and reduced, respectively, after polydatin treatment in 5-FU-R cells. Moreover, the apoptosis signals, including BAX, BAK, cleaved caspase 3, cleaved caspase 9, and cytochrome *c* were significantly elevated, implying that polydatin might affect the chemosensitivity of 5-FU-R in a way that is independent of TYMS and P53.

## 5. Conclusions

**Figure 8:**
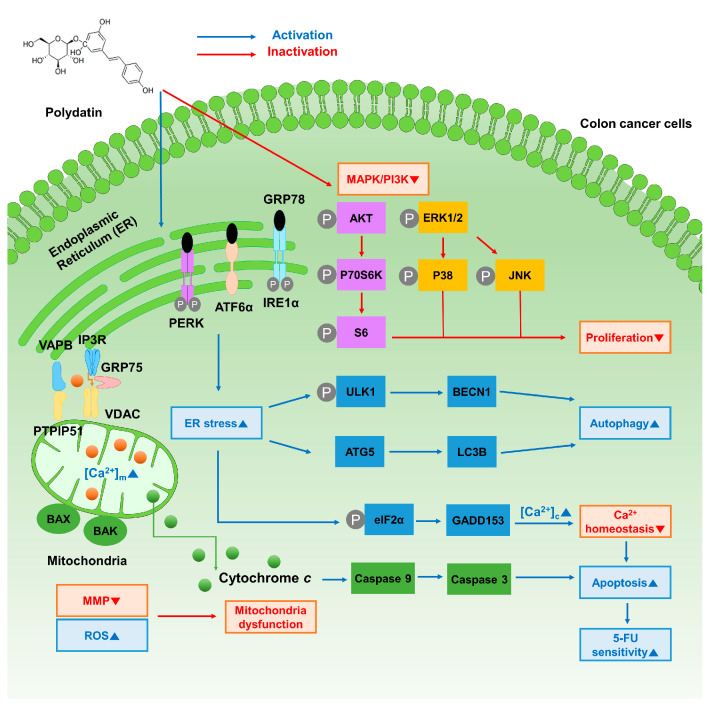
Schematic illustration of polydatin on colon cancer cells.

In conclusion, this study elucidates the mechanisms through which polydatin influences the apoptotic process and improves the drug resistance of 5-FU in colon cancer cells (Figure 8). We confirmed that polydatin suppresses the progression of colon cancer cells. Next, the mitochondrial dysfunctions in terms of membrane potential and ROS generation were determined. Moreover, the apoptotic effects of polydatin, induced by the disruption of calcium homeostasis, and the expression levels of related proteins were verified. It is concluded that the combination of polydatin with conventional anticancer agents could ameliorate the limitations of 5-FU, as polydatin enhances the chemosensitivity of colon cancer cells. Polydatin can be an innovative medical agent for the treatment of colon cancer, as demonstrated by its versatility and protective effect in various medical fields. Although our study is confined to an in vitro assessment, these results may suggest the possibility of endless application as the basis for future in vivo studies.

## Data Availability

Data is contained within the article.
